# TRAJECTORIES OF POSITIVE AND NEGATIVE AFFECT: A COORDINATED ANALYSIS OF 11 LONGITUDINAL SAMPLES

**DOI:** 10.1093/geroni/igz038.2563

**Published:** 2019-11-08

**Authors:** Emily D Bastarache, Eileen K Graham, Ryne Estabrook, Anthony Ong, Andrea Piccinin, Scott Hofer, Avron Spiro III, Daniel K Mroczek

**Affiliations:** 1 Northwestern University, Evanston, Illinois, United States; 2 Northwestern University, Chicago, Illinois, United States; 3 Cornell University, Ithaca, New York, United States; 4 University of Victoria, Victoria, B.C., United States; 5 Boston University, Boston, Massachusetts, United States

## Abstract

This study assessed age-graded change in positive and negative affect over decades of the lifespan. We conducted a coordinated integrative data analysis (IDA) using data from 11 longitudinal samples, comprising a total of 74076 respondents, spanning the ages of 11 to 106. Positive and negative affect were measured using the CES-D in 8 studies, the PANAS in 3 studies, and the MIDI scale in the MIDUS with three to eleven measurement occasions across studies. To assess and compare the extent and nature of change in affect over time across studies, analyses were coordinated, deploying identical multi-level growth models on each dataset. The curvilinear models suggested PA was best characterized by an inverted U-shaped trajectory, peaking in the mid-to-late 50s, while change in NA was best described by a U-shaped curve, bottoming out in the late 60s. We also found measure-related differences in the proportion of variance in affect attributable to within- or between person differences; The majority of the variability in CES-D-assessed affect was attributable to within-person differences over time, while the variability in PANAS-assessed affect was predominantly attributable to between-person differences. Overall, the results did not support steady improvement of emotional experience over the entire life-course as previous studies have suggested, but show promise for midlife when PA peaks and NA bottoms out. This study demonstrates the value of coordinated conceptual replications, resolving some of the mixed findings in the literature regarding age-graded change in affect and enhancing the current understanding of the longitudinal affect phenomenon.

